# Surveillance of Adverse Events Following Varicella Vaccine Immunization in Zhejiang Province, China, from 2020 to 2022

**DOI:** 10.3390/vaccines13010057

**Published:** 2025-01-10

**Authors:** Hui Liang, Xiaohua Qi, Yaping Chen, Xuejiao Pan

**Affiliations:** Institute of Immunization and Prevention, Zhejiang Center for Disease Control and Prevention, Hangzhou 310051, China; hliang@cdc.zj.cn (H.L.); xhqi@cdc.zj.cn (X.Q.); ypchen@cdc.zj.cn (Y.C.)

**Keywords:** varicella vaccine, adverse events, AEFI, surveillance, China

## Abstract

**Background**: China has a high incidence rate of varicella yet a low coverage rate of the varicella vaccine (VarV), with safety concerns being a leading cause of the lack of vaccination willingness. This study aimed to describe VarV-related adverse events following immunization (AEFIs) and analyze their characteristics in Zhejiang, China, 2020–2022. **Methods**: VarV-related AEFIs in Zhejiang Province from 1 January 2020 to 31 December 2022 were collected through the Chinese National AEFI Information System (CNAEFIS) for a descriptive epidemiological analysis. **Results**: From 2020 to 2022, a total of 1477 VarV-related AEFI cases were reported (incidence rate: 34.79/100,000). The three most frequently reported clinical symptoms of common adverse reactions were fever, redness, and induration at the vaccination site. The distribution of VarV-related AEFIs varied significantly by age, dose, severity, and season. VarV-related AEFIs were more likely to be non-severe adverse events that occurred in the summer and winter seasons following the first dose of vaccine and among those under 3 years old. The top three regions with the highest incidence rates were Lishui City (59.53/100,000), Quzhou City (41.05/100,000), and Jinhua City (40.43/100,000). Most of the cases achieved full recovery without treatment (96.21%), and the rest were successfully treated without any sequelae. **Conclusions**: VarV demonstrates a safe profile in Zhejiang Province. Most VarV-related AEFIs are common reactions without requiring treatment, and the rates of rare and severe AEFIs remain low. Consistent monitoring, investigation, and diagnosis are needed to guide future VarV research and vaccination strategy adjustment. Our findings call for more policy changes, such as integrating VarV into China’s National Immunization Program and conducting more trials to evaluate the safety and effectiveness of VarV.

## 1. Introduction

Varicella (chickenpox), caused by primary infection with varicella–zoster virus (VZV), is an acute, infectious disease commonly occurring in infancy and children [1]. VZV has only one known serotype, with humans as the only reservoir [1]. VZV is highly contagious, with a secondary attack rate ranging from 61 to 100% [2]. It mainly spreads from person to person via droplets, aerosols, or direct contact with rashes and respiratory secretions [2]. Most symptoms of varicella are self-limiting, characterized by systemic papules, pruritus, and shingles [1]. However, in severe cases, it can cause life-threatening complications such as pneumonia, encephalitis, and hemorrhagic conditions [3].

Varicella is a significant public health concern with a huge disease burden globally [4]. Each year, at least 140 million varicella cases occur around the world, including 4.2 million severe cases leading to hospitalization and 4200 deaths caused by varicella [4]. In China, the disease burden of varicella is on the rise, with an annual incidence increasing by 30% from 3/100,000 in 2005 to 70/100,000 in 2015, causing significant direct medical expenses as well as other indirect costs [5]. According to data from the National Infectious Disease Surveillance System in 2019, varicella was the third most preventable infectious disease in China, with 981,700 reported cases, second only to tuberculosis and influenza [6]. In Jiangsu Province, the reported incidence of varicella exceeded 80/100,000 during 2021–2022, ranking first among infectious disease emergencies [7]. In Zhejiang Province, a total of 144,276 varicella cases were reported from 2019 to 2022, with an annual incidence rate ranging from 47.35/100,000 to 82.80/100,000 [8]. Varicella outbreaks are concentrated in schools and childcare institutions, accounting for 30% of all public health emergencies related to infectious diseases.

The varicella vaccine (VarV) is a live attenuated VZV vaccine that has been widely recognized as the most effective measure for the prevention and control of varicella around the world [2]. According to a global meta-analysis, the vaccine effectiveness of the VarV was about 80% in a one-dose schedule and >92% in a two-dose schedule [9]. The VarV has been demonstrated to be a highly reliable and effective vaccine in curtailing varicella transmission and preventing varicella-related morbidity and mortality [7,10]. In the US, the VarV was first approved in 1995 [11] and has been widely adopted for all children in all states [12]. The universal varicella immunization program in the US has almost eliminated all varicella in children [13]. The VarV was also introduced into routine childhood immunization programs in many other countries, such as Canada, Australia, and Uruguay, significantly reducing the incidence of varicella and varicella-related morbidity [14]. The WHO recommends that the coverage rate of the VarV should exceed 80% in countries with a high disease burden of varicella [4].

In China, the VarV was introduced in 1997 to prevent varicella. However, the VarV is not included in China’s National Immunization Program, and recipients have to pay out of pocket for the vaccine [15]. With socioeconomic development and the increasing awareness of disease prevention, the public’s willingness to vaccinate against varicella has also increased [16]. However, the coverage rate of the VarV in China is still not satisfactory. According to a recent survey in China, the vaccination rate of one-dose VarV was only 67.1% (65.4~68.8%), which included 24 cities (e.g., Shanghai and Tianjin) that have incorporated the VarV into their local immunization programs [16,17]. Apart from financial restraints, safety concerns have been listed as a leading cause of the public’s lack of willingness to get vaccinated [18].

As a live attenuated vaccine, the VarV may induce adverse reactions due to continued virus replication, leading to adverse events following immunization (AEFIs). AEFIs refer to any untoward medical occurrence following immunization that does not necessarily have a causal relationship to the vaccine [19,20]. Although most AEFIs are minor and self-limiting, some may cause severe neurological and cardiac adverse events, leading to rehospitalization and even death [21,22]. Since 1995, over 200 million VarVs have been administered globally, and at least 86 fatal AEFIs have been reported following the vaccination [23,24]. The safety assessment of the VarV is crucial for improving the public’s awareness and acceptance of the VarV. Therefore, we conducted the current study to report VarV-related AEFIs and analyze their characteristics in Zhejiang, China, 2020–2022. Our findings would provide valuable insights and a scientific basis for future VarV research and vaccination strategy adjustment.

## 2. Materials and Methods

### 2.1. Data Sources

VarV-related AEFIs in Zhejiang Province from 1 January 2020 to 31 December 2022 were collected through the Chinese National AEFI Information System (CNAEFIS). The CNAEFIS is a nationwide, online AEFI surveillance system in China that provides data on vaccine safety at a national level [7]. The AEFIs are reported by authorized physicians in each vaccination clinic [25]. The CNAEFIS is managed by a professional evaluation team at the Chinese Center for Disease Control and Prevention (CDC), covering all parts of the country. The monitoring scope includes all vaccines marketed in China and the recipients, with a focus on vaccine AEFIs [25]. Information on the vaccine administration and doses during the same period was collected through the Zhejiang Province Comprehensive Management Information System for Vaccines and Immunization.

### 2.2. Surveillance of AEFI

The CNAEFIS is a passive reporting system based on voluntary reporting by authorized healthcare personnel. According to the National Monitoring Plan for Suspected Abnormal Vaccination Reactions (2022 Edition), medical institutions, vaccination units, and other organizations or personnel with reporting responsibility should report any suspected AEFI directly online to the CNAEFIS. For those who cannot report online due to limited resources, they should fill in the AEFI case report card and promptly report to the county (district, city) CDC by email, fax, etc., who will then report the case information online to the CNAEFIS. CDC at all levels investigates, reviews, and manages all reported cases through the online reporting system of the CNAEFIS. Some cases are classified by the CDC, while others are assessed by an expert committee composed of experts at the county, district, and city levels, generally including clinicians, epidemiologists, and other professionals. The expert committee conducts investigations and diagnoses according to the severity of the abnormal reactions. Any dispute over the classification will be resolved through a re-appraisal and re-examination by the expert team.

### 2.3. Definition and Classification

AEFIs were assessed and recorded up to one month following vaccination. According to the National Surveillance Plan for Suspected Abnormal Reactions to Vaccination (2022 Edition), an AEFI is defined as a reaction or event that occurs after vaccination and is suspected to be related to the vaccine [4]. After investigation, diagnosis, and analysis, the reported AEFI cases are classified into the following six categories according to the causes: common adverse reactions, rare adverse reactions, vaccine quality accidents, vaccination accidents, coincidental events, and psychogenic reactions, each defined as below:

Common adverse reactions are caused by the inherent characteristics of the vaccines themselves, which will only cause transient physiological dysfunction in the body, mainly including fever and local redness and swelling, accompanied by malaise, fatigue, and loss of appetite.

Rare adverse reactions refer to adverse reactions that damage the tissues, organs, and normal functions of recipients during or after vaccination with standard vaccines.

Vaccine quality accidents are caused by substandard vaccine quality, leading to damage to the recipient’s body tissues, organs, and functions after vaccination.

Vaccination accidents refer to adverse reactions that damage the tissues, organs, and normal functions of recipients due to violations of vaccination work specifications, immunization procedures, vaccine use guidelines, and vaccination plans.

Coincidental events refer to when the recipient is in the incubation period or prodromal stage of a certain disease at the time of vaccination, and the disease coincidentally occurs after vaccination.

Psychogenic AEFIs are individual or group reactions that occur due to psychological factors in the recipient during or after vaccination, which include anxiety, depression, sadness, insomnia, panic attacks, agitation, and fear.

In addition, we further classified AEFIs into severe and non-severe cases according to the severity of adverse events. Severe AEFIs refer to any life-threatening reactions that require hospitalization or prolonged hospitalization and may lead to persistent or significant physical disability/incapacity or even death. They also include congenital abnormalities and birth defects that are suspected to be caused by vaccination of the recipient’s mother during pregnancy [4]. Non-severe cases refer to non-life-threatening reactions that do not require hospitalization and do not cause severe adverse outcomes [4].

### 2.4. Data Collection

The live attenuated VarV is a non-national immunization program vaccine, and the vaccination plan is formulated by each province. The VarV vaccination guidelines of Zhejiang Province recommend a 2-dose vaccination schedule. The first dose should be given at 12–18 months of age, ideally at 15 months of age or older; the second dose should be given at 3 years of age or older and completed before the age of 4. This study collected all live attenuated VarV cases that reported AEFIs in Zhejiang Province from 2020 to 2022. For the present analysis, we included vaccine recipients of all ages during the predefined study period. Cases that were not directly related to the VarV were excluded. Ethical approval was obtained from the Ethics and Research Committees of Zhejiang CDC (2019-001-43, approval date: 7 July 2020). Due to the study’s observational nature and the use of a de-identified dataset, informed consent was waived by the Ethics Committee.

### 2.5. Statistical Analysis

The AEFI case data were exported to an Excel file and analyzed using R4.4.1 software. Descriptive epidemiological methods were used to analyze the reported incidence and distribution characteristics of VarV-related AEFIs. Categorical variables were reported as frequencies and proportions. We calculated the incidence rate (/100,000) as the number of AEFI reports/administered doses × 100,000. The Cochran–Armitage trend test was used to compare the reporting rates of VarV-related AEFIs by different characteristics. Bonferroni correction was used for pairwise comparisons. Statistical significance was defined as a two-sided *p*-value < 0.05.

## 3. Results

### 3.1. Overview of VarV-Related AEFIs

**Figure 1:**
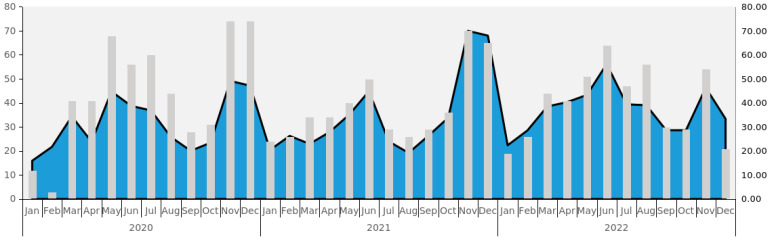
Reporting trends of AEFIs following VarV from 2020 to 2022, with peaks in summer and winter seasons (/100,000 doses). VarV: varicella vaccine; AEFIs: adverse events following immunization.

Table 1 shows an overview of the reported VarV-related AEFIs by year and category. From 2020 to 2022, there were a total of 4.2452 million doses of the VarV administered in Zhejiang Province, among which 1477 AEFI cases were reported, with an incidence rate of 34.79/100,000. The reported incidence rates by year were 33.45/100,000 in 2020, 33.50/100,000 in 2021, and 37.88/100,000 in 2022, which showed no significant differences according to the Cochran–Armitage trend test (Z = 1.929, *p* = 0.054). For occurrence time, among the 1477 AEFI cases, 1092 were reported on the day after vaccination (73.93%), 346 were reported within 1–3 days after vaccination (23.43%), and the remaining 39 were reported more than 4 days after vaccination (2.64%). The longest interval between vaccination and AEFIs was 29 days. Figure 1 shows the wave patterns of VarV-related AEFIs, which present seasonal fluctuations with two peaks in the summer and winter seasons.

For the AEFI category, no vaccine quality accident or vaccination accident was reported. The reported cases and incidence rates for common adverse reactions, rare adverse reactions, coincidental events, and psychogenic reactions were 1333 (31.40/100,000), 118 (2.78/100,000), 21 (0.49/100,000), and 5 (0.12/100,000), respectively.

There were 1333 common adverse reactions (90.25%), including 785 cases occurring after the first dose (incidence rate: 43.06/100,000) and 548 occurring after the second dose (incidence rate: 22.62/100,000), with statistically significant differences (χ^2^ = 137.76, *p* < 0.0001). In addition, there were 118 rare adverse reactions, including 76 cases occurring after the first dose (incidence rate: 4.17/100,000) and 42 occurring after the second dose (incidence rate: 1.73/100,000), with statistically significant differences (χ^2^ = 21.322, *p* < 0.0001).

### 3.2. Clinical Symptoms

Table 2 shows the various clinical symptoms of common adverse reactions of VarV-related AEFIs. The three most frequently reported reactions were fever, redness, and induration at the vaccination site, with incidence rates being 21.37/100,000, 7.02/100,000, and 0.46/100,000, respectively. Among the 907 fever cases, 542 had simple fever, 132 had fever with crying, 113 had fever with gastrointestinal symptoms (including loss of appetite, abdominal pain, diarrhea, vomiting, etc.), and 21 had fever with rash. According to the vaccine instructions, among the adverse reactions reported in the clinical trial phase, pain, transient fever, and transient rash are common adverse reactions (10%).

For rare adverse reactions, the most common reactions were allergic rash (*n* = 82) and urticaria (*n* = 19), with the incidence rate being 1.93/100,000 and 0.45/100,000, respectively. In addition, there were four cases of maculopapular rash and four cases of thrombocytopenic purpura, both with an incidence rate of 0.09/100,000. Other rare symptoms included allergic purpura, angioedema, febrile seizures, dry eye disease, headache, varicella-like rash, etc.

### 3.3. Epidemiological Distribution

Table 3 shows the distribution of VarV-related AEFIs by gender, age, dose, severity, and season. Among the total 1477 cases, 776 were male, and 701 were female, with a male-to-female ratio of 1.11:1. There were no significant gender differences in the incidence rate of VarV-related AEFIs (*p* = 0.787). Regarding age distribution, the number of VarV-related AEFIs in the age groups ≤1, 2–3, 4–7, and ≥8 years were 626, 600, 186, and 65, respectively, corresponding to incidence rates of 42.69/100,000, 35.69/100,000, 25.29/100,000, and 17.96/100,000, respectively, showing a decreasing trend with increasing age (*p* < 0.001). As for dose distribution, 760 VarV-related AEFIs occurred after the first dose, while 717 occurred after the second dose. The incidence rate of VarV-related AEFIs was significantly higher following the first dose than the second dose (41.69/100,000 vs. 29.60/100,000, *p* < 0.001). Regarding severity distribution, most were non-severe cases (*n* = 1466), accounting for 99.26% of all cases, and only 12 were severe cases (0.81%). The incidence rate of VarV-related AEFIs was significantly higher in non-severe cases than in severe cases (34.53/100,000 vs. 0.28/100,000, *p* < 0.001). As for seasonal distribution, the number of VarV-related AEFIs in spring, summer, autumn, and winter was 229, 445, 349, and 454, respectively, corresponding to an incidence rate of 26.50/100,000, 38.69/100,000, 28.90/100,000, and 44.37/100,000, respectively, with significant seasonal differences (*p* < 0.001).

Table 4 shows the distribution of VarV-related AEFIs by location. The top three regions with the largest number of VarV-related AEFI cases were Hangzhou City (*n* = 301), Jinhua City (*n* = 247), and Ningbo City (*n* = 240). The top three regions with the highest incidence rate of VarV-related AEFIs were Lishui City (59.53/100,000), Quzhou City (41.05/100,000), and Jinhua City (40.43/100,000).

### 3.4. Severe Cases and Outcomes

A total of 12 severe AEFIs were reported, with an incidence rate of 0.28/100,000. Table 5 shows the details of the 12 severe AEFIs, such as age, gender, classification, and clinical diagnosis. Ten cases occurred after the first dose of vaccination, with an incidence of 0.55/100,000, while the remaining two cases occurred after the second dose, with an incidence rate of 0.08/100,000, and the difference was statistically significant (χ^2^ = 6.4268, *p* = 0.011). Most severe AEFIs occurred in males (9/12, 75%) and at the age of 3 and below (11/12, 91.67%). Five cases had received other vaccines concomitantly with the VarV (5/12, 41.67%), mainly group A and group C meningococcal polysaccharide conjugate vaccine (MCV-AC). Three cases occurred on the day of vaccination (3/12, 25%), while the other nine cases occurred between 1 day and 29 days after vaccination (9/12, 75%). After investigation and diagnosis by the expert committee, seven cases were determined to be rare adverse reactions (7/12, 58.33%), and five cases were classified as coincidental events (5/12, 41.67%). The most common clinical diagnosis was thrombocytopenic purpura (6/12, 50%), and other clinical diagnoses included febrile seizure (*n* = 2), acute upper respiratory infection (*n* = 1), thrombocytopenia (*n* = 1), allergic purpura (*n* = 1), and allergic rash (*n* = 1). An illustrative description of each case can be found in Supplementary File S1.

Most of the cases achieved full recovery without requiring any treatment (*n* = 1421, 96.21%), and 3.79% of the cases improved after treatment (*n* = 56). No cases had sequelae.

## 4. Discussion

Varicella is a highly contagious infectious disease primarily affecting school-age children [1]. Although most symptoms of varicella are mild, varicella can lead to serious complications such as pneumonia, encephalitis, meningitis, etc. [1]. Varicella remains highly prevalent in China, causing significant healthcare costs and disease burden [5]. The most effective way to prevent varicella is through VarV vaccination, yet the VarV is not included in China’s National Immunization Program [15]. This study evaluated the safety of the VarV by describing the incidence of VarV-related AEFIs and analyzed the epidemiological distributions across various characteristics in Zhejiang Province from 2020 to 2022.

Our results showed that the incidence of VarV-related AEFIs was 34.79/100,000 doses. The three most frequently reported clinical symptoms of common adverse reactions following VarV vaccination were fever, redness, and induration at the vaccination site. The distribution of VarV-related AEFIs varied significantly by age, dose, severity, and season. VarV-related AEFIs were more likely to be non-severe adverse events that occurred in the summer and winter seasons following the first dose of vaccine and among those under 3 years old. The top three regions with the highest incidence rate were Lishui City, Quzhou City, and Jinhua City. A total of 12 severe AEFIs were reported, with an incidence rate of 0.28/100,000. Most of the cases achieved full recovery without treatment, and the rest were successfully treated without any sequelae. Our results generally support the effectiveness and safety of VarV in Zhejiang Province.

Our study showed that the reported incidence rate of VarV-related AEFIs in Zhejiang Province from 2020 to 2022 was 34.79/100,000 doses, with no statistical significance by year. This rate was lower than the reported 52.7/100,000 doses in the US from 1995 to 2005 based on the Vaccine Adverse Event Reporting System (VAERS) [26] but slightly higher than the reported 30.6/100,000 doses in the US from 2006 to 2020 [27]. However, it is much higher than the reported 3.71/100,000 doses in Japan between 2005 and 2015 based on the post-marketing reporting system [28]. This discrepancy may be explained by the different reporting systems in various countries, such as different definitions, assessments, observation timeframes, reporting responsibilities, punishment mechanisms, and reporting procedures [7,26]. In addition, it may also be related to the cultural and sociodemographic differences across countries. For instance, most Chinese families have only one child after three decades of the one-child policy as the national policy [29]. Therefore, the parents attach great importance to their only child and are especially sensitive to any potential adverse reactions related to the vaccines, leading to a high reporting rate. Woodward et al. [30] conducted a comprehensive review of the VarV safety profile worldwide over 22 years and found that VarV-related AEFIs have decreased from 50/100,000 doses in 1995 to 4/100,000 doses in 2016, which was much lower than the incidence reported in our study. However, it should be noted that the global estimate was based on diverse reporting sources, including spontaneous post-marketing and non-interventional study reports, which may lead to high heterogeneity in various countries and incomplete or missing information in low-reporting countries. Another explanation may be that the global number of vaccination doses increased much faster than the number of AEFI reports, leading to a relatively smaller reported incidence rate. Furthermore, previous studies were conducted at earlier times before our study when the reporting systems may not have developed fully, thus leading to lower reporting rates than our study.

Compared with other parts of China, the incidence rate of VarV-related AEFIs in our study was lower than the reported 39.74/100,000 doses in Guangxi Province from 2020 to 2022 [31] and the reported 47.54/100,000 doses in Jiangsu Province from 2015 to 2023 [7]. However, this rate was higher than the reported national level of 29.79/100,000 doses from 2014 to 2020 [32] and 22.91/100,000 doses from 2021 to 2022 [33]. Similar to other vaccines, 1/3–3/4 of the national AEFI surveillance cases in recent years have been reported from the eastern regions. This may be due to the larger population, higher number of vaccinations, more comprehensive surveillance network coverage, and higher surveillance sensitivity in the eastern regions. In addition to the characteristics of the vaccine, factors such as parents’ awareness of vaccine reactions may also affect the incidence of AEFI reports. In sum, these findings show that the incidence of AEFIs following the VarV is within the expected range with mild reactions, confirming the safety of the VarV.

The reported VarV-related AEFIs were predominantly common adverse reactions, accounting for 90.25% of all cases. The most commonly reported symptoms included fever, redness, and swelling at the vaccination site. Rare adverse reactions, such as allergic rash, thrombocytopenic purpura, febrile convulsions, and allergic purpura, were rarely reported, indicating that VarV vaccination is relatively safe. Most of the VarV-related AEFIs occurred within 1 day after vaccination. It is recommended that parents should pay close attention to children’s reactions within the first 30 min observation window and strengthen home care on the day of vaccination to detect and respond to any abnormalities in a timely manner. In addition, health inquiries before vaccination should be strengthened to reduce the occurrence of coincidental cases as much as possible. In addition, all reported AEFI cases showed a good prognosis, and no serious sequelae were found. In sum, our findings showed that the VarV was safe when used in a large population in Zhejiang Province.

The epidemiological distribution of VarV-related AEFIs in our study was similar to the patterns in other studies. The reported incidence rates were almost equally distributed by gender, with no statistically significant difference between males and females. For age distribution, VarV-related AEFIs were mainly concentrated in those aged under 3 years old (84.01%), which was in line with the existing VarV vaccination procedures in Zhejiang Province [4]. Regarding dose distribution, the incidence of reported VarV-related AEFIs was much higher following the first dose than the second dose, consistent with previous studies [7,34]. This may be related to the younger age and less developed immune systems in those receiving the first doses of the VarV, making them more vulnerable to AEFIs [34,35,36]. Another explanation may be that parents pay more attention to their children during their first exposure to the VarV and are more likely to report any abnormal reactions and seek professional help [35,36]. For seasonal distribution, the highest incidence of VarV-related AEFIs occurred in winter, which aligned with the peak outbreak of varicella in winter, leading to increased vaccinations and higher vigilance towards adverse reactions [37]. Another peak occurred in summer, which may be related to increased skin exposure in hot weather, leading to increased reports of rashes, redness, and swelling. Regarding regional distribution, the incidence of VarV-related AEFIs varied greatly across cities in Zhejiang Province, ranging from 22.62/100,000 doses to 59.53/100,000 doses. This may be related to the different levels of emphasis on AEFIs and training on reporting in various cities [38]. It is necessary to strengthen surveillance and reporting training to improve the investigation capabilities and diagnostic skills of all monitoring personnel.

This study has several limitations. First, our data came from the CNAEFIS based on passive surveillance, and the accuracy of the report may be affected by the monitoring and reporting quality of the surveillance system. Second, some cases reported rashes, but the number, shape, and duration of the rashes were not described. Third, we did not collect detailed information about the VarV vaccine manufacturers and thus could not compare VarV-related AEFIs based on different production processes and companies, which might affect safety profiles. Fourth, we could not explore further specific etiologies of AEFIs, such as neurological, cardiac, or allergic reactions, which were unavailable from the reporting system. Finally, traditional monitoring analysis has some limitations, and advanced big data mining technology can quickly and accurately identify vaccine adverse reactions, which should be explored further.

## 5. Conclusions and Recommendations

In conclusion, the VarV demonstrates a safe profile in a large population in Zhejiang Province from 2020 to 2022. Most VarV-related AEFIs are common reactions without requiring further treatment, and the rates of rare and severe AEFIs remain low. Consistent monitoring, investigation, and diagnosis are needed to guide future VarV research and vaccination strategy adjustment. It is recommended that the VarV be integrated into China’s National Immunization Program to improve VarV coverage and prevent varicella-related adverse outcomes. In the future, more studies are needed to assess the disease burden of varicella and evaluate the accessibility, safety, cost-effectiveness, and government budget of the VarV.

In 2020, the WHO approved the Immunization Agenda 2030 (IA2030), emphasizing the establishment of a world where people of all ages can fully benefit from vaccines [39]. As of 2021, 51 countries among the 194 WHO member states and regions have included the varicella vaccine in their National Immunization Programs [39]. Although existing studies show that the VarV has good efficacy and tolerability, reports of adverse reactions, such as varicella-like rash and thrombocytopenic purpura, have also aroused public concern [40]. In addition, self-paid vaccines and uneven economic development in various regions may further affect the public’s vaccination willingness.

In recent years, many cities, such as Shanghai and Jiangsu, have successively incorporated the VarV into their local immunization programs. Zhejiang, as an area with a high incidence of varicella, should also evaluate the rationality of incorporating the VarV into the local immunization plan. A sufficient evidence base is crucial to guide scientific vaccination strategies and management, thus reducing vaccine inequalities caused by vaccine literacy and economic development and ultimately improving vaccination coverage and protecting more children from preventable infectious diseases.

## Figures and Tables

**Table 1:** Reported VarV-related AEFIs in Zhejiang Province by year and category from 2020 to 2022.

	Common Adverse Reaction		Rare Adverse Reaction		Coincidental Event		Psychogenic Reaction		Total	
Year	No.	Incidence Rate *	No.	Incidence Rate	No.	Incidence Rate	No.	Incidence Rate	No.	Incidence Rate
2020	462	29.05	57	3.58	11	0.69	2	0.13	532	33.45
2021	426	30.82	34	2.46	3	0.22	0	0.00	463	33.50
2022	445	34.97	27	2.12	7	0.55	3	0.24	482	37.88
Total	1333	31.40	118	2.78	21	0.49	5	0.12	1477	34.79

VarV: varicella vaccine; AEFIs: adverse events following immunization; * /100,000 doses.

**Table 2:** Distribution of clinical symptoms of common adverse reactions after VarV vaccination in Zhejiang Province from 2020 to 2022.

Symptoms	No.	Incidence Rate
Fever		
37.1–37.5	57	0.13
37.6–38.5	365	0.86
≥38.6	485	1.14
Redness		
≤2.5	44	0.10
2.5–5	162	0.38
5	92	0.22
Induration		
≤2.5	8	0.02
2.5–5	61	0.14
≥5	54	0.13
Crying	197	0.46
Weakness	85	0.20
Lethargy	15	0.04
Loss of appetite	93	0.22
Nausea	14	0.03
Dizziness/headache	15	0.04
Rash/itch	21	0.05
Muscle pain	4	0.01
Vomit	41	0.10
Sweating	11	0.03
Paleness	9	0.02
Abdominal pain/diarrhea	6	0.01
Other	13	0.03

VarV: varicella vaccine.

**Table 3:** Epidemiological distribution of AEFIs following VarV from 2020 to 2022 in Zhejiang Province.

Variables		AEFI Cases		Incidence Rate (/100,000) *	*p*
		No.	Proportion (%)		
Gender	Male	776	52.54	35.05	0.7866
	Female	701	47.46	34.51	
Age	≤1	626	42.38	42.69	<0.0001
	2–3	600	40.62	35.69	
	4–7	186	12.59	25.29	
	≥8	65	4.40	17.96	
Dose	1	760	51.46	41.69	<0.0001
	2	717	48.54	29.60	
Severity	Serious	12	0.81	0.28	<0.0001
	Non-serious	1466	99.26	34.53	
Seasons	1–3	229	15.50	26.50	<0.0001
	4–6	445	30.13	38.69	
	7–9	349	23.63	28.90	
	10–12	454	30.74	44.37	

* Incidence rate was calculated as the number of AEFI reports/administered doses × 100,000

**Table 4:** Distribution of VarV-related AEFIs by location.

Regions (City)	2020		2021		2022		Total	
	No.	Incidence Rate	No.	Incidence Rate	No.	Incidence Rate	No.	Incidence Rate
Hangzhou	130	34.06	91	29.92	80	29.43	301	31.43
Jiaxing	32	26.63	36	31.80	35	36.51	103	31.29
Huzhou	15	24.43	24	33.78	22	24.22	61	27.32
Ningbo	82	36.42	73	37.42	85	48.35	240	40.27
Wenzhou	50	24.99	51	28.43	59	36.66	160	29.60
Shaoxing	29	24.82	19	20.33	20	22.14	68	22.62
Jinhua	93	39.73	72	35.93	82	46.46	247	40.43
Quzhou	19	44.58	18	47.52	10	29.40	47	41.05
Zhoushan	3	17.61	4	23.86	5	31.79	12	24.23
Taizhou	51	37.32	50	41.69	43	39.61	144	39.44
Lishui	28	51.00	25	49.21	41	78.56	94	59.53
Total	532	33.45	463	33.50	482	37.88	1477	34.79

VarV: varicella vaccine; AEFIs: adverse events following immunization.

**Table 5:** Details of 12 severe cases of VarV-related AEFIs.

No.	Age	Gender	Vaccination Time	AEFI Occurrence Time	Co-Administration with other Vaccines	Classification by Cause	Clinical Diagnosis
1	1	F	19 September 2022	After 26 days	PCV13	Coincidental events	Thrombocytopenic purpura
2	1	M	9 March 2022	After 1 day	No	Coincidental events	Febrile seizure
3	2	M	5 March 2022	After 12 days	No	Rare adverse reactions	Thrombocytopenic purpura
4	3	F	17 August 2022	After 29 days	MCV-AC	Rare adverse reactions	Thrombocytopenic purpura
5	1	M	29 April 2020	After 9 days	No	Rare adverse reactions	Thrombocytopenic purpura
6	1	M	15 June 2020	The same day	No	Rare adverse reactions	Febrile seizure
7	3	M	28 September 2020	After 4 days	No	Coincidental events	Thrombocytopenic purpura
8	3	F	2 December 2020	The same day	MCV-AC	Coincidental events	Acute upper respiratory infection
9	3	M	13 October 2020	After 13 days	MCV-AC	Rare adverse reactions	Thrombocytopenic purpura
10	1	M	15 December 2020	After 5 days	No	Coincidental events	Thrombocytopenia
11	6	M	22 July 2021	After 16 days	No	Rare adverse reactions	Allergic purpura
12	3	M	31 August 2022	The same day	MCV-AC	Rare adverse reactions	Allergic rash

VarV: varicella vaccine; AEFIs: adverse events following immunization; PCV13: pneumococcal vaccine 13; MCV-AC: group A and group C meningococcal polysaccharide conjugate vaccine.

## Data Availability

Data cannot be shared openly due to the protection of patient information and privacy. Access to the data is subject to approval and a data-sharing agreement with Zhejiang CDC.

## Supplementary Materials

The following supporting information can be downloaded at: https://www.mdpi.com/article/10.3390/vaccines13010057/s1.
