# Supplementary material for: Surveillance of Adverse Events Following Varicella Vaccine Immunization in Zhejiang Province, China, from 2020 to 2022
Source: Vaccines (Basel). 2025 Jan 10;13(1):57. doi: 10.3390/vaccines13010057 (PMC11769265; doi:10.3390/vaccines13010057)
Supplement: Supplementary file 1 [file vaccines-13-00057-s001.zip › vaccines-3385827-supplementary.pdf]

Detailed illustration of the 12 severe cases of VarV-related AEFIs

Case 1: The patient developed bleeding spots on her skin at home two hours after vaccination without obvious inducement. The bleeding sports present with a pinpoint-like appearance, mainly around the orbit. The patient had no epistaxis, gum bleeding, hematemesis, melena, hematuria, fever, cough, or vomiting. The patient had thick stools 3-4 times a day without mucus, pus, or blood. The platelet count was 27×10^9/L↓. The patient was given symptomatic and supportive treatment such as hemostatic injections, vitamin C injections, and venous globulin injections.

Case 2: The child developed crying and restlessness at about 5 am the day after vaccination, accompanied by seizures. The symptoms included loss of consciousness, eyes turning upward, cyanosis of the lips, trismus, and limb rigidity, which lasted for about 1 minute and then resolved on its own. The patient was sent to the County Maternity Hospital for treatment at about 8:30 am, and his body temperature reached a maximum of 39°C. He had seizures again around noon, and the head CT scan showed no apparent abnormalities. The patient was treated with Cefdinir granules, Xiaoerguqiaoqingshao granules, and the compound Lactobacillus acidophilus. The parents also prepared Motrin.

Case 3: The patient received the VarV vaccine on March 5. On March 17, he developed pinpoint-like bleeding spots on his eyelids, left earlobe, and instep, which gradually subsided without special treatment. On March 23, he had scattered petechiae all over the body. The patient was diagnosed with thrombocytopenic purpura, which was successfully treated with hormones and gamma globules.

Case 4: The patient developed black bruises on the lower limbs 29 days after vaccination. There were many pinpoint-sized bleeding spots and blue-gray ecchymoses on the trunk and limbs, which did not fade under pressure. The platelet count was 20×10^9/L↓. After admission to the hospital, he was given 15g of G-globulin (9.17-9.18) and an intravenous infusion of hemostatic drugs to prevent bleeding. Head CT and abdominal B-ultrasound showed no abnormality, and the patient improved spontaneously.

Case 5: The patient had bleeding spots, multiple ecchymoses all over his body, and a small number of nosebleeds. The blood platelet count was 2×10^9/L. He was diagnosed with thrombocytopenic purpura and was hospitalized. During hospitalization, a 10g human immunoglobulin injection was given intravenously, and symptomatic treatment was given.

Case 6: On the day after vaccination, the patient had a fever of 38.0°C at noon and was given an oral antipyretic drug (the name of the drug was unknown). At 9 pm, his body temperature rose to 39.0°C, and he developed febrile seizures. He was immediately sent to the city's childcare hospital for treatment. His body temperature dropped below 37.0°C. He had a normal spirit but a poor appetite. He no longer had any seizures.

Case 7: The patient received the VarV vaccine at 8:22 am on September 28. On October 2, he had a few bleeding spots on both lower limbs. On October 4, he had multiple ecchymoses on both lower limbs. On October 6, the patient was sent to Jinhua Maternal and Child Health Hospital for a check-up. The blood routine showed that platelets were 20*10^9/L, and the blood routine gradually returned to normal after being given G-globulin.

Case 8: The patient developed the following symptoms two hours after the vaccination: the corners of the mouth suddenly twitched, with foam at the mouth and the eyes rolling up, and she was nonresponsive. The symptoms lasted for about ten minutes. She was sent to the emergency department of the Maternal and Child Health Hospital and completed relevant examinations. Her body temperature was measured at 38°C. She was given ceftriaxone 1g qd (12.2-12.5) intravenously for anti-infection. Discharge diagnosis: 1. Acute upper respiratory infection, seizures to be investigated: febrile seizures? Epilepsy? Cerebral palsy?

Case 9: The patient had bleeding spots on the skin of the trunk 13 days after vaccination. He had no fever, headache, vomiting, cervical lymphadenopathy, and no abnormality in the stool. On October 28, he had increased bleeding spots. He was visiting Shanghai with his parents at that time, and he was hospitalized at the Children's Hospital of Fudan University in Shanghai. The initial diagnosis was thrombocytopenic purpura, which has been cured.

Case 10: The patient had bleeding spots on his skin five days after the vaccination and was sent to the Affiliated Children's Hospital for treatment. The platelet count was 6*109/L, and the discharge diagnosis was immune thrombocytopenia. During hospitalization, he was given Milosone to suppress the immune response, omeprazole to protect his stomach, and oral prednisone acetate 18.75 mg bid (12.21--12.22).

Case 11: The patient developed scattered red rashes on the inner thighs and ankles 16 days after vaccination, which then developed to the back, buttocks, abdomen, and arms. On August 1,

he was sent to the Children's Hospital of Zhejiang University School of Medicine and was diagnosed with allergic purpura.

Case 12： The patient developed rashes on the day of vaccination, and the outpatient diagnosis was "anaphylaxis." He was given an intramuscular injection of 0.13 mg epinephrine and oral administration of loratadine syrup. After a few minutes, the rashes gradually subsided, and the patient had itching skin, occasional crying, and a relatively stable mood.

Abbreviations: VarV: Varicella vaccine; AEFIs: adverse events following immunization
